# 2‐Hydroxyethyl Methacrylate, Methyl Methacrylate and Ethyl Acrylate Sensitisation: A 7‐Year Retrospective Study

**DOI:** 10.1111/cod.70054

**Published:** 2025-11-18

**Authors:** Mira Jagodich, Dominik Németh, Adrienn Tóth, Zsuzsanna Zsófia Szalai, Györgyi Pónyai

**Affiliations:** ^1^ Department of Dermatology Heim Pál National Pediatric Institute Budapest Hungary; ^2^ Department of Dermatology, Venereology and Dermato‐Oncology Semmelweis University Budapest Hungary; ^3^ Doctoral College Semmelweis University Budapest Hungary

**Keywords:** 2‐hydroxyethyl methacrylate, artificial nails, contact allergy, ethyl acrylate, methyl methacrylate, patch test

## Abstract

**Background:**

Acrylates are increasingly common contact allergens; sensitisation is frequently associated with nail products. No prior study has been found in the European literature in which both methyl methacrylate (MMA) and ethyl acrylate (EA), in addition to 2‐hydroxyethyl methacrylate (2‐HEMA) were included in the standard baseline series.

**Objectives:**

To investigate the prevalence and characteristics of 2‐HEMA, MMA and EA sensitisation at a university centre in Hungary.

**Methods:**

This retrospective study included 2005 consecutive patients who underwent patch testing between 2018 and 2024 with the European baseline series (EBS) extended with 2‐HEMA (in 2018), MMA and EA.

**Results:**

Out of the tested 2005 patients, 74 (3.7%) were positive for at least one of the three acrylates; among them 86.5% were women. Mostly the hands (70.3%) and the face (24.3%) were symptomatic. Nail product exposure was significantly more frequent among younger patients. 20.3% of the acrylate‐positive patients had no hypersensitivity to 2‐HEMA, only to MMA and/or EA. Reactions to 2‐HEMA and EA mostly appeared on D2, whereas MMA first reactions were predominantly on D7.

**Conclusion:**

Our findings suggest that testing MMA and EA in the EBS would be beneficial. Evaluating patch tests until the 7th day is essential.

## Graphical abstract

In our cohort acrylate hypersensitivity was associated with using of artificial nail products (mainly young women). Reading the test until the 7th day helps to avoid missing delayed reactions (mostly by MMA). Testing 2‐HEMA exclusively would have resulted a loss of 20% of the sensitisations, it is worth testing 2‐HEMA + MMA and EA to improve diagnostic accuracy.

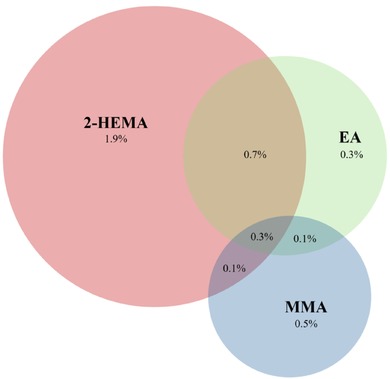

## 1 Introduction

Acrylates are synthetic, thermoplastic resins, widely used across various industrial, medical, and cosmetic applications. Polymerisation is a chemical reaction of monomers and their corresponding esters, occurring either spontaneously, under light emitting diode, ultraviolet (UV) light, using chemical initiators, accelerators or heat, etc. The monomers have significant allergic potential, in contrast to the polymers, which are inert substances [1, 2, 3, 4].

In recent years, acrylates have emerged as significant and increasingly prevalent causes of both occupational and non‐occupational allergic contact dermatitis. Artificial nail products are commonly responsible for the increased prevalence of acrylate contact allergy [2]. Allergic contact dermatitis from wearing artificial nails is associated mostly with 2‐hydroxyethyl methacrylate (2‐HEMA), methyl methacrylate (MMA) and ethyl acrylate (EA) sensitization [5].

These acrylates are widely used also in healthcare products, and can be found in dental prostheses and filling compounds, dentures, bone cement, orthopaedic prostheses, surgical glues, wound dressings, electrocardiogram electrodes, transcutaneous electrical nerve stimulators, glucose sensors, insulin pumps, hearing aids, contact and intraocular lenses. They also occur in cosmetic/aesthetic products: dermal fillers, glue (false eyelashes, hair extensions and fixation of wigs and hairpieces, nails cosmetics), consumer products (cleaning products, polishes, waxes, disposable diapers, incontinence pads, textiles, fitness devices). Acrylates have been used for decades in the industry as gluing and sealing material; they can be found also in computer disks, fibreglass, glues, paints, plastic products, sealants [1, 2, 4, 6, 7, 8, 9, 10]. Owing to their widespread use and strong sensitising potential, the American Contact Dermatitis Society (ACDS) named acrylates Contact Allergen of the Year in 2012 [11].

This single‐centre retrospective study aimed to assess the 7‐year trend of positive patch test reactions to 2‐HEMA, MMA and EA in consecutive patients referred for patch testing, and to evaluate whether the inclusion of MMA and EA in the European baseline series (EBS) would provide diagnostic benefit.

## 2 Materials and Methods

### 2.1 Patch Testing and Data Collection

Over a 7‐year period (1 January 2018–31 December 2024), 2005 consecutive patients were patch tested with the European baseline series (EBS) at the Allergology Outpatient Unit of the Department of Dermatology, Venereology and Dermato‐oncology, Semmelweis University. Besides 2‐HEMA, the standard environmental series at our department contains MMA and EA as well. HEMA 2% in petrolatum (pet.), MMA 2% pet. and EA 0.1% pet. were used. Patch testing was performed using Smart Practice Europe allergEAZE preparations (SmartPractice Europe, Greven, Germany).

The antigens were applied on the patient's asymptomatic back in 48 h occlusion. Reactions were evaluated in the first 20–60 min (immediate‐type reaction), and after 48 h occlusion on days (D) 2, D3, D4 and D7. The evaluation of the reactions was performed in accordance with the ESCD guideline [12]. For analysis, the peak reaction strength—defined as the highest recorded score across all evaluating days—was used to represent the overall intensity of each patient's reaction to a given allergen.

The frequency of 2‐HEMA, MMA and EA contact hypersensitivity was investigated along with the co‐occurrence of the three allergens. Sensitisation was defined as a positive patch test reaction on any evaluating day to at least one acrylate allergen. Demographic (age and sex), occupational (sources of exposure) and clinical data (localisation of skin symptoms) of sensitised patients were retrospectively collected from our institutional database.

A positive patch test reaction was considered relevant in case of reported exposure to acrylates, including the use of artificial nail products, or occupations involving potential acrylate exposure (nail technicians, cosmetologists, workers using chemical products).

### 2.2 Statistical Analysis and Data Management

Patient and examination characteristics were summarised descriptively. Continuous variables are presented as mean ± standard deviation (SD); categorical variables as frequencies and percentages. Normality of distribution was assessed using the Shapiro–Wilk test. Group comparisons of non‐normally distributed or small‐sample continuous data were performed with the Mann–Whitney *U* test. Associations between continuous or ordinal variables were evaluated with the Spearman rank‐correlation coefficient. Comparisons of categorical variables between independent patient subgroups were performed using the Chi‐square test. Demographic and clinical characteristics of sensitised patients were explored with the MOAHLFA index. Two‐tailed *p*‐values less than 0.05 were considered statistically significant. All statistical analyses were performed using IBM, SPSS, v30.

## 3 Results

### 3.1 Patient Population

**FIGURE 1:**
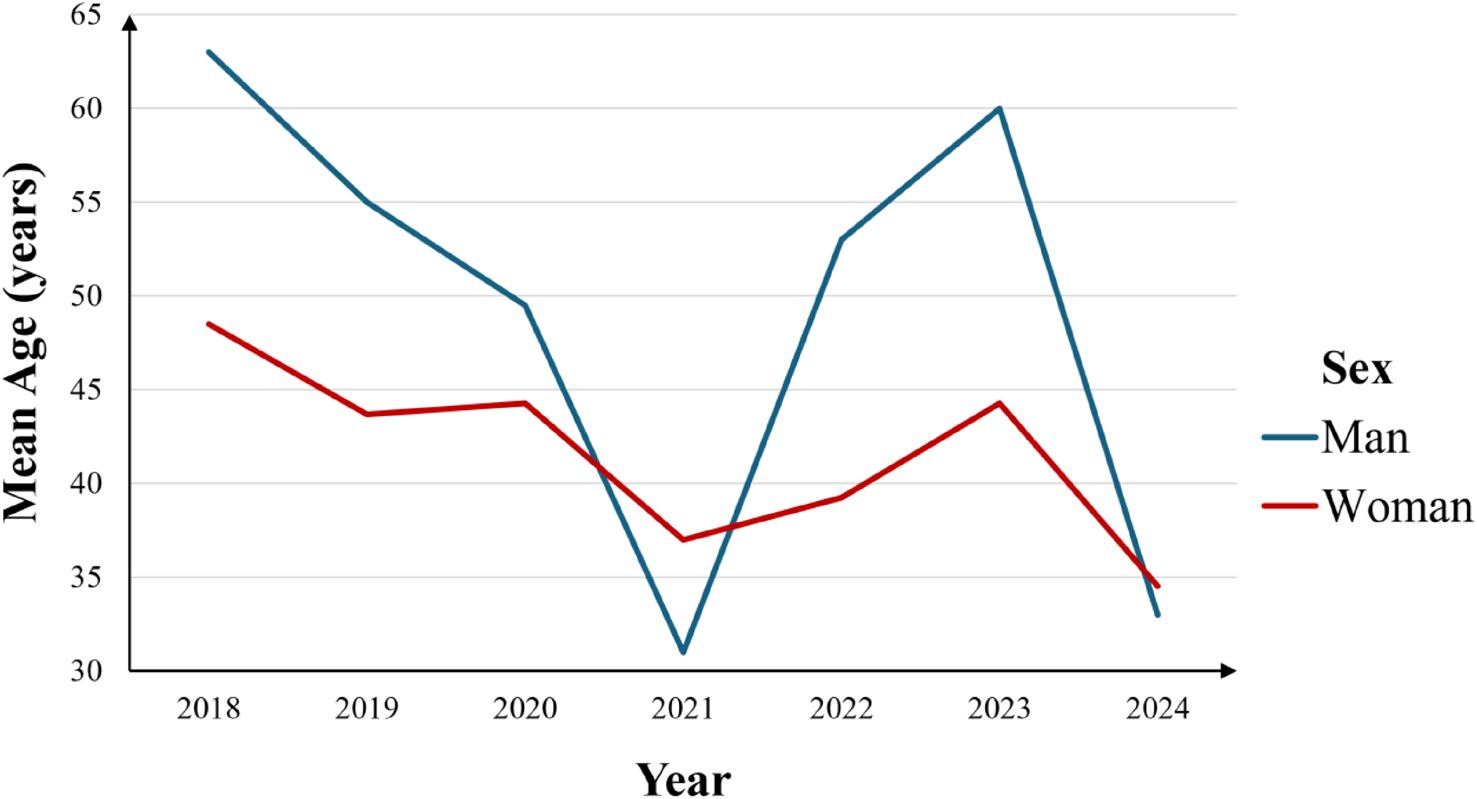
Age of patients with acrylate hypersensitivity by year.

Out of the 2005 examined, 74 (overall prevalence: 3.7%) patients were positive for at least one of the three acrylates. Among them, 64 were women, with a mean age of 41.68 ± 14.22 years (range 17–73 years). Relevant positive reactions were recorded in 45 patients (overall prevalence: 2.2%), of whom 41 were women (91.1%). Women showed a higher rate of relevant positive reactions than men (64.1% and 40%, respectively). The mean age of women with positive and relevant positive reactions (40.72 ± 14.14 years and 39.66 ± 13.16 years, respectively) was lower, than that of men (47.80 ± 13.91 years and 48.25 ± 18.79 years, respectively). A significant negative association between the year of testing and age among sensitised patients (Spearman *r* = −0.25, *p* = 0.031) was found, indicating that sensitised individuals tended to be younger in more recent years (Figure 1).

### 3.2 Acrylate Hypersensitivity by Year

**FIGURE 2:**
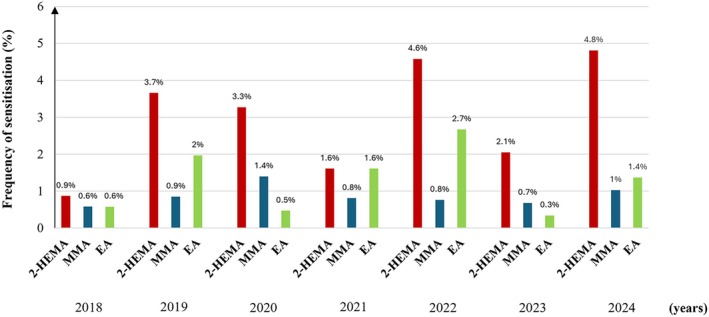
Yearly prevalence of 2‐HEMA, MMA, and EA sensitization between 2018 and 2024. (EA, Ethyl acrylate; MMA, Methyl methacrylate; 2‐HEMA, 2‐hydroxyethyl methacrylate).

Sensitisation to at least one of the acrylates and relevant positive reactions was most frequently observed in 2022 and 2024 (Table 1). Among the three tested acrylates, 2‐HEMA hypersensitivity was overall the most frequently detected over the 7‐year period (*p* < 0.001), with 59 positive cases (2.9%), compared to 26 cases for EA (1.3%) and 17 cases for MMA (0.8%). The yearly distribution of acrylate sensitisation was analysed separately for each allergen (Figure 2). Differences in 2‐HEMA sensitisation prevalence over the study years were observed (*p* = 0.027). The annual prevalence increased from 0.9% in 2018 to 4.8% in 2024, suggesting an increasing trend over time, although the linear‐by‐linear association did not reach statistical significance (*p* = 0.051). No significant differences or trends were found for MMA (*p* = 0.971 and *p* = 0.817, respectively) or EA (*p* = 0.127 and *p* = 0.774, respectively).

**TABLE 1:** Prevalence of acrylate contact hypersensitivity and relevant reactions by years.

Year	Positive reactions*	%	Relevant reactions**	%
2018	5/343	1.5	2/343	0.6
2019	16/355	4.5	11/355	3.1
2020	9/214	4.2	7/214	3.3
2021	6/248	2.4	3/248	1.2
2022	14/262	5.3	10/262	3.8
2023	8/292	2.7	2/292	0.7
2024	16/291	5.5	10/291	3.4
Total	74/2005	3.7	45/2005	2.2

*Note*: *, ** Data is presented as the number of positive reactions* or relevant positive reactions*/the total number of tested patients each year.

### 3.3 Co‐Sensitisation

**FIGURE 3:**
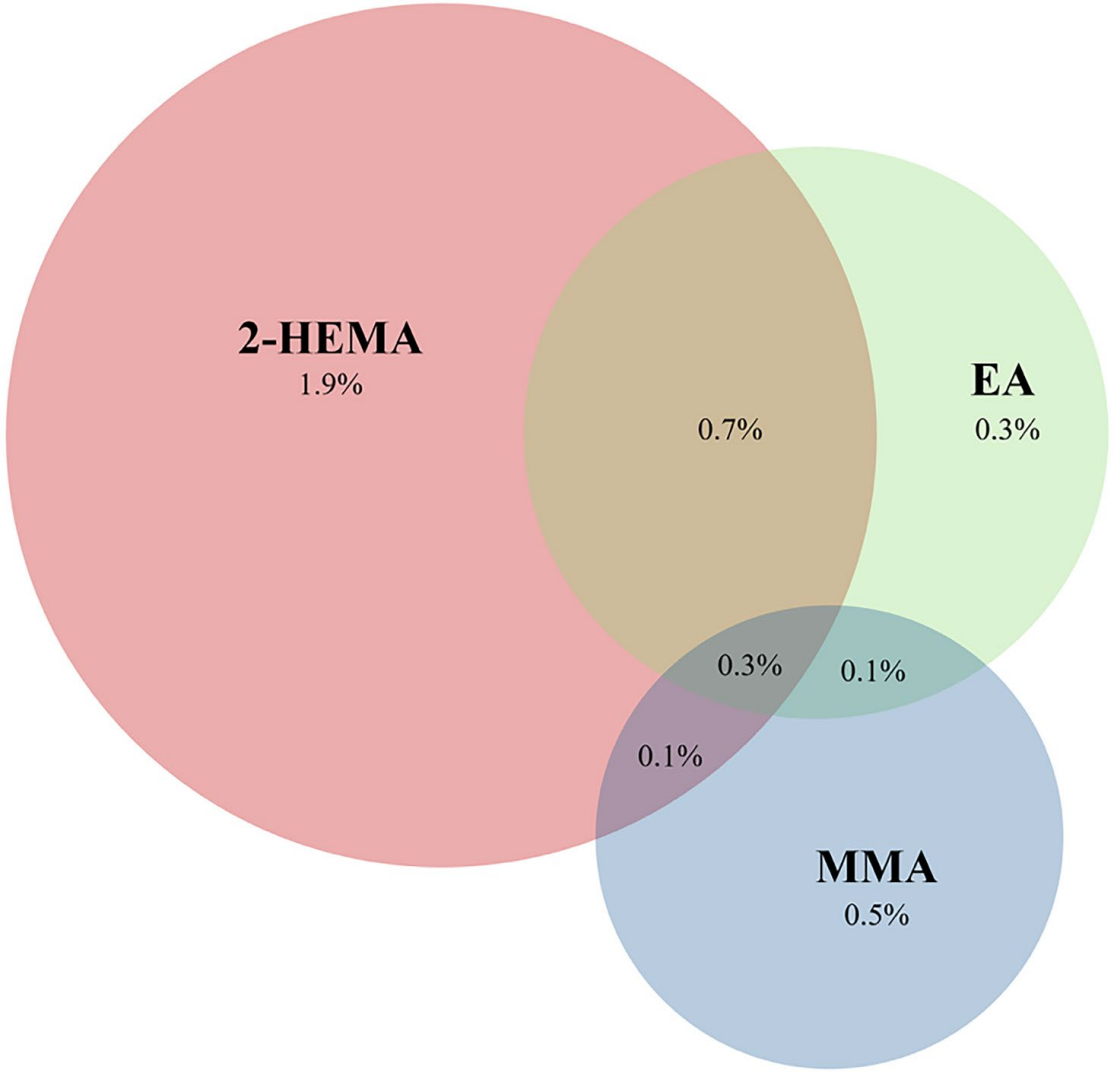
Venn diagram illustrating the overall prevalence of contact hypersensitivity to 2‐hydroxyethyl methacrylate (2‐HEMA), ethyl acrylate (EA), and methyl methacrylate (MMA) among the tested population (*n* = 2005). Percentages represent the proportion of the total cohort sensitized to each individual acrylate or specific allergen combinations.

Overall prevalence of sensitisation was 2.9% for 2‐HEMA, 0.8% for MMA and 1.3% for EA. 20 out of the 26 EA sensitised patients exhibited concomitant 2‐HEMA hypersensitivity. MMA sensitisation occurred as a standalone reaction in 9 patients, compared to 8 patients with co‐sensitisation. Notably, among the total 74 sensitised patients, 10 (13.5%) with MMA sensitisation and 6 (8.1%) with EA sensitisation had a negative reaction to 2‐HEMA. Among these cases one patient (1.4%) showed sensitisation to both MMA and EA without concomitant 2‐HEMA reaction (Table 2 and Figure 3).

**TABLE 2:** Acrylate contact hypersensitivity and co‐sensitization per year.

Allergens	Tested years							Total	%
	2018	2019	2020	2021	2022	2023	2024		
2‐HEMA only	1	8	6	2	6	5	10	38	1.9
MMA only	2	1	2	0	0	2	2	9	0.5
EA only	0	1	0	2	2	0	0	5	0.3
2‐HEMA + MMA	0	0	0	0	1	0	0	1	0.1
2‐HEMA + EA	2	4	0	0	4	1	3	14	0.7
MMA + EA	0	1	0	0	0	0	0	1	0.1
2‐HEMA + MMA + EA	0	1	1	2	1	0	1	6	0.3
Total	5	16	9	6	14	8	16	74	3.7

Abbreviations: 2‐HEMA, 2‐hydroxyethyl methacrylate; EA, ethyl acrylate; MMA, methyl methacrylate.

### 3.4 Strength of Reaction

**FIGURE 4:**
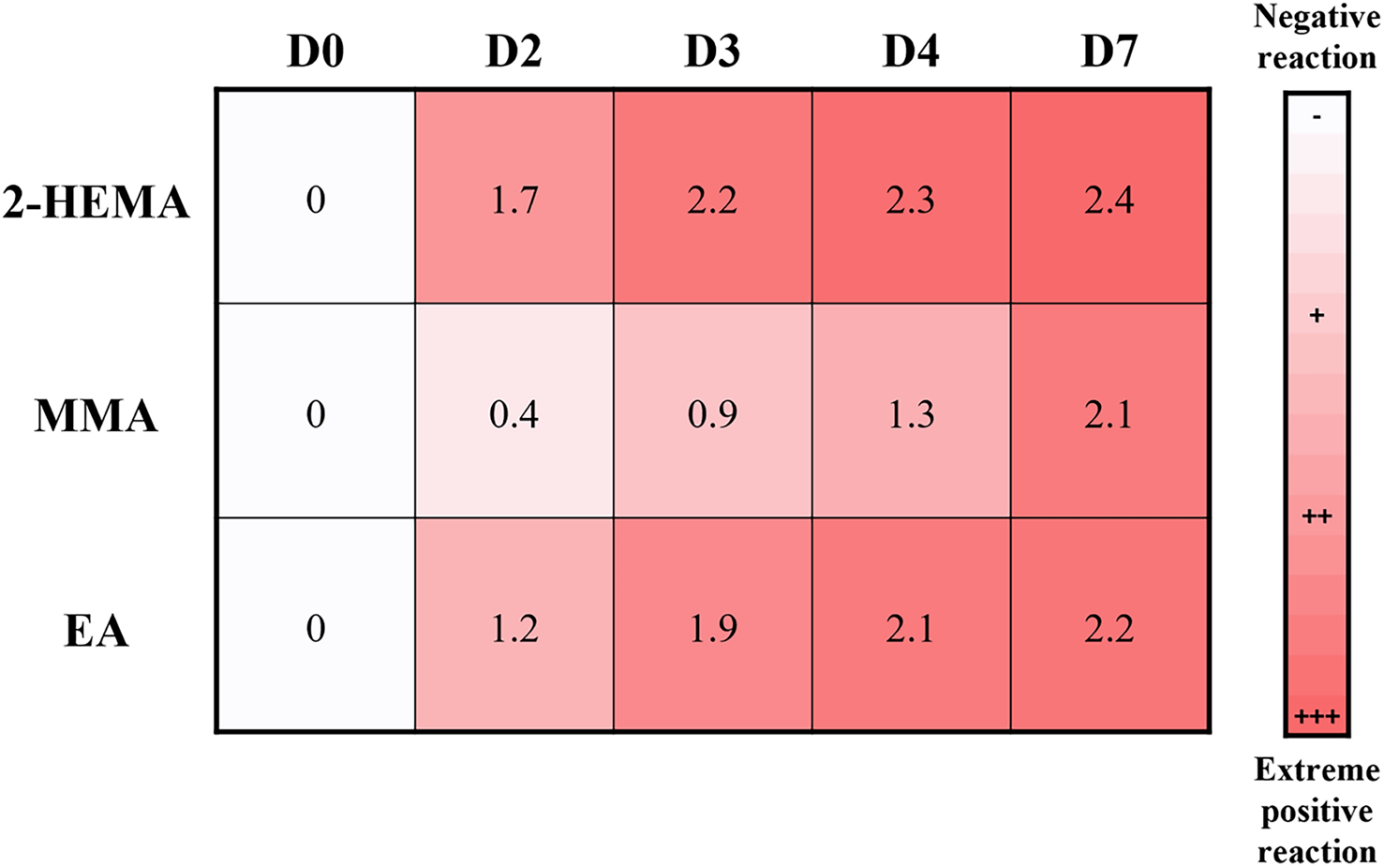
Heatmap illustrates the average patch test reaction strength across all sensitised patients to each acrylate over the five reading days. (D, Day; EA, Ethyl acrylate; MMA, Methyl methacrylate; 2‐HEMA, 2‐hydroxyethyl methacrylate).

All three acrylates demonstrated an increasing trend in reaction strength over evaluating days (Figure 4). Among the 59 patients sensitised to 2‐HEMA, 24 (40.7%) exhibited strong (+++) peak reactions, while the remaining showed moderate (++) responses. In contrast, the majority of peak reactions to MMA and EA were moderate (++), observed in 88.2% and 80.8% of cases, respectively. Notably, patients co‐sensitised to all three acrylates (2‐HEMA, MMA, and EA) had significantly stronger 2‐HEMA reactions compared to those sensitised to 2‐HEMA only (*p* = 0.006).

### 3.5 Appearance of First Positive Reactions

**FIGURE 5:**
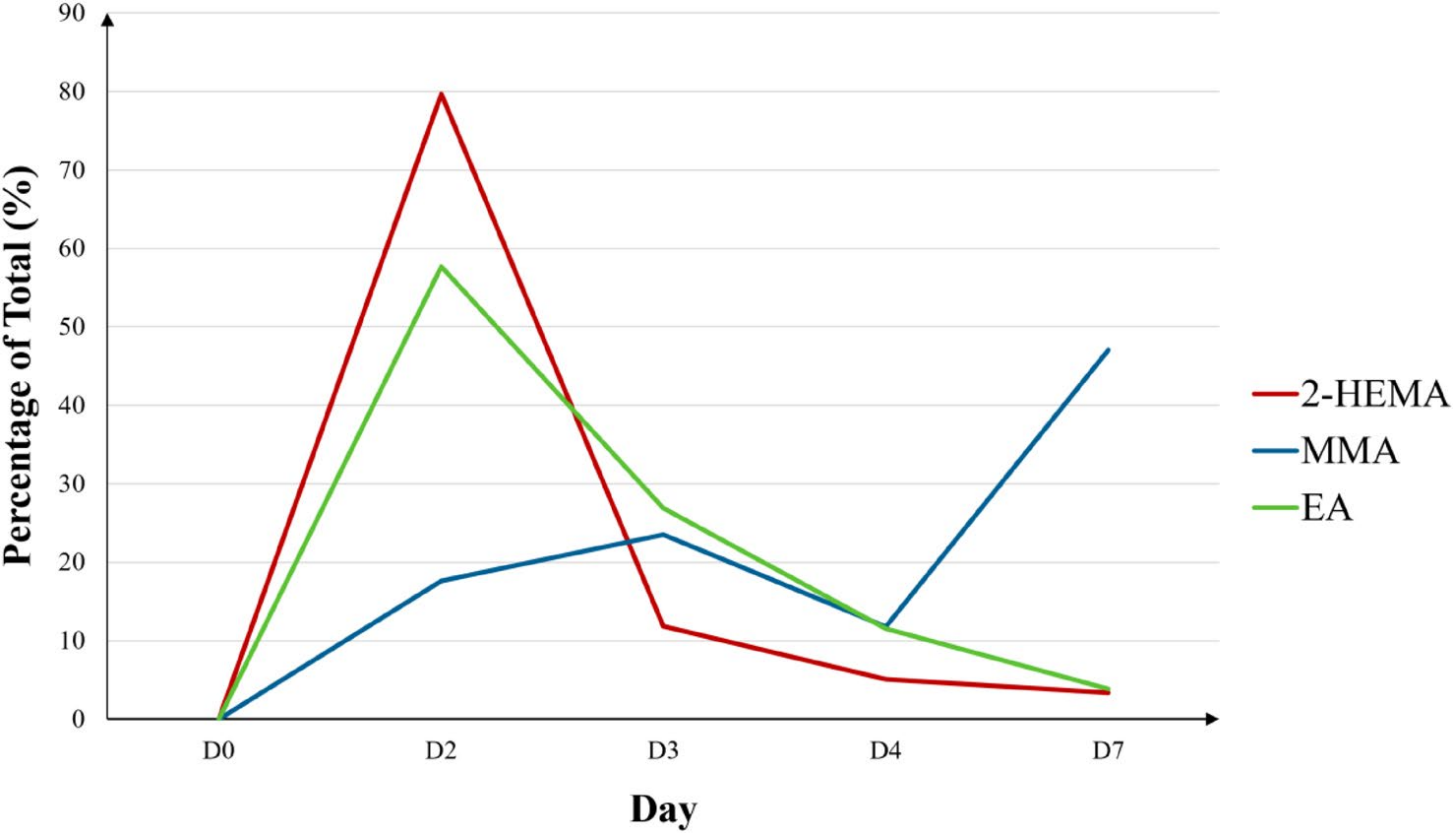
Line chart showing the distribution of first positive reactions over time as a percentage of total sensitizations per acrylate. (D, Day; EA, Ethyl acrylate; MMA, Methyl methacrylate; 2‐HEMA, 2‐hydroxyethyl methacrylate).

No immediate‐type hypersensitivity reactions were observed for any of the three tested acrylates. The distribution of first positive reaction days significantly differed among the acrylates (*p* < 0.001). Reactions to 2‐HEMA and EA most commonly appeared on D2, whereas MMA reactions were predominantly observed on D7. Although all three acrylates elicited first positive reactions on D7, the frequency was significantly higher for MMA compared to 2‐HEMA and EA (*p* < 0.001) (Table 3 and Figure 5).

**TABLE 3:** The appearance of the first positive reactions by acrylates.

	D0^a^	D2^b^	D3^b^	D4^b^	D7^b^
2‐HEMA	0	47/59 (79.7%)	7/59 (11.9%)	3/59 (5.1%)	2/59 (3.4%)
MMA	0	3/17 (17.7%)	4/17 (23.5%)	2/17 (11.8%)	8/17 (47.1%)
EA	0	15/26 (57.7%)	7/26 (26.9%)	3/26 (11.5%)	1/26 (3.9%)

Abbreviations: 2‐HEMA, 2‐hydroxyethyl methacrylate; D, day; EA, ethyl‐acrylate; MMA, methyl‐methacrylate.

^a^ D0: immediate‐type reactions.

^b^ Data is presented as the number of first positive reactions on the specific day/the total number of patients sensitised to the acrylate (%).

### 3.6 Nail Products and Occupation

**FIGURE 6:**
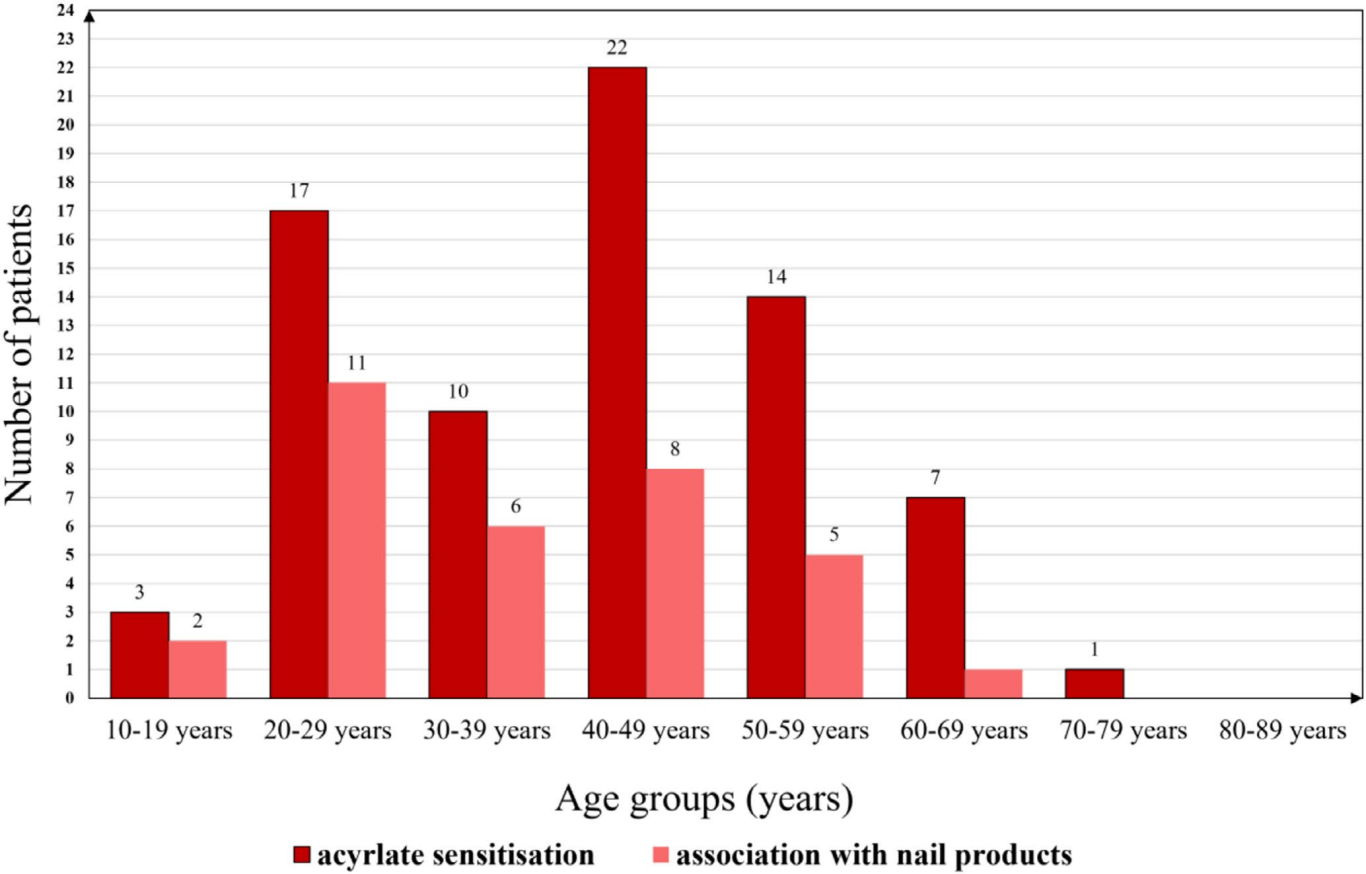
Age distribution of acrylate contact hypersensitivity and association with artificial nail products.

Nail product exposure was significantly more frequent among sensitised patients under 40 years of age compared to those aged 40 or older (*p* < 0.01) (Figure 6). 30/74 (40.5%) of the sensitised patients were younger than 40 years old, and 19 cases (63.3%) in this group were associated with wearing artificial nail products and/or being a nail technician. 44/74 (59.5%) were aged 40 or older; in their group only 14/44 of the cases (31.8%) were associated with wearing or using artificial nail products and/or being a nail technician. An increased vulnerability was observed in the younger population: from the 3 female patients under the age of 20 years, 2 (66.7%) had artificial nails.

From the 45 patients with relevant acrylate allergy 33 (73.3%) were attributed to artificial nails. Besides them, we encountered factory workers and factory/chemical engineers (13.3%), nurses (6.7%), a beautician (2.2%) and a cleaner (2.2%) who had come into contact mainly with industrial adhesives, medical adhesives, eyelash adhesives, or cleaning supply and other adhesives (e.g., surface treatment products, surface cleaners) during their daily work.

The exposure was occupational in 25/59 cases (42.4%) with 2‐HEMA, in 4/17 cases (23.5%) with MMA and in 13/26 cases (50%) with EA hypersensitivity.

### 3.7 Skin Symptoms by Body Parts, MOAHLFA Index

Out of the 74 sensitised patients, 34 (45.9%) had simultaneous involvement of more than one body part, and 11 (14.9%) showed widespread skin symptoms affecting more than three anatomical areas. The hands were the most typically involved area, followed by the head and/or face (Table 4). The MOAHLFA index was applied to the study population (Table 5).

**TABLE 4:** Skin symptoms by localisation.

Localisation	No. of patients (%)
Widespread^a^	11 (14.9)
Head and/or face	18 (24.3)
Neck	3 (4.1)
Chest	3 (4.1)
Back	1 (1.4)
Arm	7 (9.5)
Hands	52 (70.3)
Thigh	1 (1.4)
Leg and/or knee	3 (4.1)
Feet	8 (10.8)

^a^ (more than 3 body parts).

**TABLE 5:** MOAHLFA index in 74 patients with positive reaction to any of the three tested acrylates.

	Positive patch test reaction to 2‐HEMA (*n* = 59)	Positive patch test reaction to MMA (*n* = 17)	Positive patch test reaction to EA (*n* = 26)
Male	4 (6.8%)	5 (29.4%)	2 (7.7%)
Occupational	25 (42.4%)	4 (25.5%)	13 (50%)
Atopy	3 (5.1%)	3 (17.7%)	1 (3.9%)
Hand	49 (83.1%)	8 (47.1%)	22 (84.6%)
Leg	7 (11.9%)	2 (11.8%)	1 (3.9%)
Face	11 (18.6%)	4 (23.5%)	7 (26.9%)
Age > 40	34 (57.6%)	11 (64.7%)	13 (50%)

Abbreviations: 2‐HEMA, 2‐hydroxyethyl methacrylate; EA, ethyl acrylate; MMA, methyl methacrylate.

## 4 Discussion

This single‐centre retrospective study examined a 7‐year trend in acrylate sensitization among 2005 consecutively patch‐tested patients at the Allergology Outpatient Unit of the Department of Dermatology, Venereology and Dermato‐oncology, Semmelweis University, Budapest. Our findings indicate a 3.7% overall prevalence of sensitisation to at least one of the three acrylates included in our extended baseline series: 2‐HEMA, MMA or EA. Specifically, the prevalence of sensitization was 2.9% for 2‐HEMA, 0.8% for MMA and 1.3% for EA. The majority of patients sensitised to MMA or EA also exhibited concomitant 2‐HEMA hypersensitivity, underscoring the diagnostic value of 2‐HEMA as a central component among acrylates in the baseline series. However, reliance on 2‐HEMA testing alone would have failed to detect one in five acrylate sensitisation cases, suggesting that using 2‐HEMA as a sole screening marker might be insufficient. Interestingly, patients co‐sensitised to all three acrylates had significantly stronger 2‐HEMA reactions compared to those sensitised to 2‐HEMA alone. This finding supports the clinical utility of MMA and EA testing, particularly in patients presenting with strong 2‐HEMA reactions. Moreover, first positive reactions to MMA were commonly observed on day 7, emphasising that the evaluation of the patch test until D7 is necessary.

2‐HEMA has been part of the European baseline series (EBS) since January 2019 [13]. In the United States, it was included in the North American Contact Dermatitis Group (NACDG) baseline series in 2007 [7]. Our findings are consistent with prior international studies reporting a growing prevalence of 2‐HEMA sensitisation, ranging from 1.5% to 3.4%, with a clear predominance among women and strong associations with nail products [13, 14, 15, 16, 17, 18, 19, 20, 21]. For instance, a one‐year Italian multicentre study (November 2017–October 2018) found a 1.5% prevalence of 2‐HEMA sensitisation among 4025 patients, with 86.9% of the cases occurring in women. The majority of the patients had strong (++) or extreme positive (+++) reactions, and 2‐HEMA sensitivity was mainly attributed to (meth)acrylates in nail products [14]. Similarly, in the United Kingdom, 15 dermatology centres included 2‐HEMA in the extended baseline patch test series during 2016–2017. From 5920 patch‐tested patients, 102 (1.7%) had a positive reaction to 2‐HEMA [15]. Other European studies report comparable rates: 1.6% in Italy (2018) [16], 2.4% in Denmark (2017–2019) [17], and 3% in the Netherlands (2019–2023), where 89.8% of the 2‐HEMA sensitised patients were female, and nail products were the most frequent cause of allergic contact dermatitis (66%) [13]. In the European Union, the prevalence of 2‐HEMA hypersensitivity was 2.3% in 13 different countries between January 2019 and December 2020 [18]. In Italy in a 5‐year period among 7133 patch‐tested patients, 147 (2.1%) had a positive reaction to 2‐HEMA with an increasing trend from 2019 (1.6%) to 2023 (2.7%). Artificial nails were the leading source of exposure [22].

2‐HEMA has been part of the EBS since 2019, but according to our best knowledge, this is the first study in Europe which evaluated all three acrylates (2‐HEMA, MMA, EA) in parallel. In contrast, the baseline series of the NACDG contains not only 2‐HEMA, but also MMA and EA as well [2]. According to the NACDG patch test data from 2007 to 2022, the prevalence of MMA sensitisation ranged from 0.7% to 1.4%, and the prevalence of EA sensitisation ranged from 0.7% to 1.7% [19, 20], results which are in line with the findings of the present study. However, these publications did not provide data on the co‐occurrence of the three acrylates, the strength of the reactions, the demographic, occupational, clinical data and the association of the contact hypersensitivity with the wearing of artificial nail products.

In the cases of acrylate hypersensitivity, it is particularly difficult to determine whether the sensitisation is occupational or not. Nail technicians, for instance, not only work with acrylate‐containing products but often also wear artificial nails themselves, making it difficult to determine the primary source of exposure. Nonetheless, in our cohort, 42.4% of 2‐HEMA, 23.5% of MMA and 50% of EA sensitised patients had identifiable occupational exposure in the medical history. The hands were the most commonly affected area, followed by head and/or face. This distribution is likely related to exposure from nail products, which are directly applied to the hands and can result in prolonged skin contact. Subsequent transfer to the face through hand‐to‐face contact may explain the frequent involvement of facial skin (ectopic dermatitis). Notably, nail product exposure was significantly more frequent among sensitised patients under 40 years of age compared to those aged 40 or older. In a retrospective analysis based on the NACDG database from 2001 to 2016, the most common contact allergens in connection with nail care products were 2‐HEMA, MMA, EA, ethyl‐2‐cyanoacrylate, and tosylamide. Most reactions to 2‐HEMA, MMA and EA were associated with wearing artificial nails, to ethyl‐2‐cyanoacrylate with nail adhesives and to tosylamide with using nail polish. (Meth)acrylate sensitised patients mostly presented with hand dermatitis [5].

Nail technicians, nail artists and consumers have an increased risk of developing (meth)acrylate contact hypersensitivity, because of the direct contact with the monomers before polymerisation. The correct technique for preparing acrylate‐containing nails is one of the most important factors to reduce the risk of sensitization [1, 5, 23]. According to the EU Cosmetics Regulation (EC 1223/2009) in November 2020, the use of 2‐HEMA in nail cosmetics has been restricted only for professional use [18]. In our cohort, acrylate sensitisation was particularly frequent among younger individuals exposed to nail products, highlighting the emerging risk associated with cosmetic use.

Our study had limitations that should be acknowledged. First, as a retrospective analysis, exposure histories were based on patient‐reported data and may be incomplete or subject to recall bias. Second, we did not perform chemical analysis of patients' personal care or occupational products, limiting the ability to definitively confirm sources of exposure. Third, demographic data were only collected for sensitised individuals; therefore, comparisons between sensitised and non‐sensitised groups could not be performed. Additionally, the single‐centre design may limit the generalisability of our findings to other populations or geographic regions.

To summarise, our results support previous international data on acrylates as increasingly common environmental contact allergens. In our cohort, women were predominantly affected, and in a significant proportion of the cases, especially in younger age groups, sensitisation was associated with exposure to artificial nail products. In accordance with that, the hands and the face were the most commonly affected regions. Additionally, our findings support the evaluation of the patch test until the 7th day to avoid missing delayed reactions, especially in the case of MMA sensitisation. Notably, exclusively relying on 2‐HEMA testing would have failed to detect 20% of the sensitisation cases. In conclusion, we observed that it was worth patch testing MMA and EA beside 2‐HEMA to improve diagnostic accuracy and clinical management.

## Conflicts of Interest

The authors declare no conflicts of interest.

## Data Availability

The data that support the findings of this study are available from the corresponding author upon reasonable request.
